# A Unified Map of Airway Interactions: Secretome and Mechanotransduction Loops from Development to Disease

**DOI:** 10.3390/arm93060051

**Published:** 2025-11-12

**Authors:** Crizaldy Tugade, Jopeth Ramis

**Affiliations:** 1 Open University System, Graduate School Department, Polytechnic University of the Philippines, A. Mabini Campus, Anonas Street, Sta. Mesa, Manila 1016, Philippines; crizaldytugade@gmail.com; 2 Department of Chemical Engineering, Technological Institute of the Philippines, Quiapo, Manila 1001, Philippines; 3 Department of Biomedical Engineering, Batangas State University—Alangilan Campus, Golden Country Homes, Brgy. Alangilan, Batangas City 4200, Philippines

**Keywords:** mesenchymal stromal cell, airway epithelial cell, airway smooth muscle cell, chondrocytes, secretome, mechanobiology, cell–cell communication

## Abstract

**Highlights:**

**What are the main findings?**

- First closed-loop model mapping bidirectional secretome–mechanotransduction feedback in airways, where mechanical cues trigger cytokine release and vice versa through YAP/TAZ signalling.
- A novel cellular specialization framework defining epithelial cells as environmental activators, smooth muscle as mechanical actuators, and chondrocytes as calcium-dependent regulators in airway homeostasis.

**What is the implication of the main finding?**

- Enables targeted therapy of airway diseases by interrupting pathological feedback loops (e.g., YAP/TAZ-mediated feed-forward stiffness traps in asthma/COPD).
- Provides a system-based framework for airway tissue engineering by incorporating mechanotransduction feedback loops essential for functional airway constructs.

**Abstract:**

Human airways maintain homeostasis through intricate cellular interactomes combining secretome-mediated signalling and mechanotransduction feedback loops. This review presents the first unified map of bidirectional mechanobiology–secretome interactions between airway epithelial cells (AECs), smooth muscle cells (ASMCs), and chondrocytes. We unify a novel three-component regulatory architecture: epithelium functioning as environmental activators, smooth muscle as mechanical actuators, and cartilage as calcium-dependent regulators. Critical mechanotransduction pathways, particularly YAP/TAZ signalling and TRPV4 channels, directly couple matrix stiffness to cytokine release, creating a closed-loop feedback system. During development, ASM-driven FGF-10 signalling and peristaltic contractions orchestrate cartilage formation and epithelial differentiation through mechanically guided morphogenesis. In disease states, these homeostatic circuits become pathologically dysregulated; asthma and COPD exhibit feed-forward stiffness traps where increased matrix rigidity triggers YAP/TAZ-mediated hypercontractility, perpetuating further remodelling. Aberrant mechanotransduction drives smooth muscle hyperplasia, cartilage degradation, and epithelial dysfunction through sustained inflammatory cascades. This system-level understanding of airway cellular networks provides mechanistic frameworks for targeted therapeutic interventions and tissue engineering strategies that incorporate essential mechanobiological signalling requirements.

## 1. Introduction: Gaps in Mapping Airway Interactomes

Despite their relative architectural simplicity, the human airways have a profoundly important role in the maintaining lung function. Upon inhalation, the airways are not only used as a pathway of air to the lungs but they also (1) improve the quality of the air by humidification and heating, (2) serve as a barrier to the external environment, (3) produce mucous to aid ejection of foreign particles and bacteria through coughing, and (4) provide structural support [1]. Each of the cell types present within the airways has a crucial role in one or more of these functions of the airways. Compromise of airway function results in a variety of pathological conditions including asthma, chronic obstructive pulmonary disease (COPD), Acute Respiratory Distress Syndrome (ARDS) and cystic fibrosis, which leads to increased mortality, morbidity, and reduces the quality of life of patients diagnosed with such diseases. These acute and chronic lung diseases currently have no single cure due to multiple contributions to the development of the disease; thus, the need to improve system-level understanding of the interactomes may elucidate how these diseases develop and could lead to potentially developing a multi-faceted treatment or cure.

Clinically, lung dysfunction manifests through two primary physiological patterns: obstructive and restrictive disorders [2]. Obstructive airway diseases, exemplified by asthma and chronic obstructive pulmonary disease (COPD), are characterised by airflow limitation on spirometry, with asthma showing bronchodilator reversibility while COPD demonstrates persistent fixed obstruction [2,3]. These conditions represent heterogeneous phenotypes that are distinguished by features such as bronchodilator response, eosinophil counts, and inflammatory patterns, which guide both management strategies and prognosis [3]. Restrictive lung diseases, in contrast, are characterised by reduced lung volumes and compliance, limiting the lung’s ability to expand during inspiration.

Efforts to resolve airway damage by trauma and disease has inspired researchers to engineer the organ itself, and over the past decade, various methodologies have been employed in vivo. Unfortunately, however, long-term success has thus far been elusive, with tissue engineered airway constructs mostly failing in animal models [4]. These failures have likely been caused by mechanical failure, inflammation, malacia, or stenosis [5,6,7,8,9]. Long-term viability of tissue-engineered airway constructs has been reported in dogs [10] and humans [11], although controversies have arisen over the scientific integrity of the trials [12]. The direct mechanisms of why these failures occurred are still unclear. Many tissue-engineered tracheas have been reported to be similar to native airways in terms of mechanical strength, glycosaminoglycan and hydroxyproline content, matured epithelial lining, and other histological aspects [13,14,15]. However, it has become clear that a major dissimilarity between these engineered airways and native airways is the absence of airway smooth muscle (ASM) in the engineered constructs. Molecular and mechanical cross-talk between cells is often fundamental to tissue growth and homeostasis [16,17,18]; therefore, the lack of ASM cells within the engineered airways could have profound effects on cellular functions that compromise airway integrity.

In this review, we aim to bring together published studies to unravel the complex interactions occurring between the cells comprising human airways and discuss their potential contribution to the maintenance of airway function.

### Anatomic Scope of the Review

To establish clear anatomical and technical contexts for this review, we define key terms as they relate to cellular interactions and mechanotransduction in airway biology.

Airways in this review encompass the conducting structures from trachea to bronchi that serve as conduits for air to the lungs, emphasising their multifunctional role in humidification, heating, barrier protection, mucus production for particle ejection, and structural support. Our focus centres on the complex cellular architecture comprising airway epithelial cells (AECs), airway smooth muscle cells (ASMCs), chondrocytes, and mesenchymal stem cells (MSCs) from the central airways (trachea and bronchi) that collectively maintain airway homeostasis through coordinated cellular interactions.

For anatomic clarity, the cartilaginous support of the airways follows a precise anatomical gradient that is critical for understanding tissue engineering applications. The trachea is supported by 16–20 horseshoe-shaped (C-shaped) hyaline cartilage rings connected by annular ligaments anteriorly and laterally, with a posterior smooth muscle (trachealis) membrane [19]. This rigid hyaline cartilage serves as the major load-bearing component of the tracheal wall [20]. Moving distally, the main bronchi maintain substantial cartilaginous support, but the cartilage progressively decreases in both quantity and structural prominence through successive bronchial generations [20]. In the smaller bronchi, cartilage appears as irregular plates rather than complete rings, while bronchioles (airways < 1 mm diameter) completely lack cartilaginous support, relying instead on smooth muscle and elastic fibres for structural integrity [20]. This proximal-to-distal cartilage gradient directly influences the mechanical properties and engineering requirements for different airway segments.

Lungs are referenced as the ultimate destination for airway-conducted air, with particular emphasis on their inherently mechanical nature—constantly subjected to stretching and relaxation during breathing cycles. This review focuses on how lung mesenchymal stem cells contribute to airway homeostasis and how mechanical processes are critical for normal lung development and regeneration following surgical interventions.

Tissue engineering in this context refers to systematic efforts to resolve airway damage from trauma and disease through the creation of functional airway constructs that incorporate essential cellular components and mechanotransduction pathways. Our review addresses the current limitations where engineered constructs often fail due to mechanical issues, inflammation, malacia, or stenosis, with a critical gap being the absence of airway smooth muscle (ASM) in many engineered airways. We propose a systems-based framework for airway tissue engineering that incorporates bidirectional secretome–mechanotransduction feedback loops, utilising mesenchymal stem cells as global mediators capable of differentiation into specific airway cell types while contributing to immune regulation and regeneration.

## 2. Airway Cell Specialisation in a Feedback Loop Model

The human airway is composed of several different types of cells, including chondrocytes, airway smooth muscle cells (ASMC), airway epithelial cells (AEC), mesenchymal stromal cells (MSC), fibroblasts, neurons, adipocytes, and endothelial cells. This review acknowledges the heterogeneity of cell types within the population, specifically the AECs, which has subphenotypes, including ciliated cells, goblet and club cells, and basal cells. The AECs discussed therein is a collective term for all its subpopulations.

Single-cell RNA sequencing studies have revolutionised our understanding of airway cellular heterogeneity and intercellular communication networks. Recent comprehensive analyses have identified previously unknown epithelial cell subtypes, characterised their distinct secretory profiles, and mapped ligand–receptor interactions between airway cell populations [21,22]. These studies reveal that mechanical perturbations reshape epithelial transcriptional states, altering paracrine signalling to mesenchymal and immune cells [23]. In addition, critical recognition of cellular heterogeneity based on developmental origins is essential for understanding differential injury responses in airway mechanotransduction networks. Airway basal cells from distinct developmental lineages—such as ventral cartilage-associated versus dorsal smooth muscle-associated regions—maintain different transcriptional signatures and mechanosensitive pathway activation patterns that directly influence their capacity for self-renewal, differentiation, and secretome production following tissue damage [24]. This developmental origin-dependent heterogeneity extends to chondrocytes, smooth muscle cells, and epithelial subtypes, creating spatially distinct repair programmes where YAP/TAZ signalling, TRPV4 channel activation, and cytokine release patterns vary significantly based on cellular developmental history. For the purposes of this review, we will focus on interactions between general cell types of chondrocytes, ASMC, AEC, and MSC.

Chondrocytes are the resident cells of cartilage and produce abundant extracellular matrix (ECM) components made up of collagens, glycosaminoglycan, and proteoglycans to create a hyaline cartilage [25,26], which mechanically supports the overall structure of the airway. Lung MSCs are capable of differentiating into chondrocytes, ASMCs, or AECs to replace cells and maintain airway homeostasis [27]. ASMCs form together a muscular tissue with connective fibres attached to the cartilage, capable of contracting the airway luminal diameter, which could modulate air resistance as it travels in and out of the lungs [28]. AECs are located on the luminal side of the airways and provide a barrier to the external environment. These cells release mucous, which traps particles, pathogens, and other foreign components for ease of ejection through coughing or sneezing [29]. Central to airway defence is the mucociliary clearance system, a coordinated mechanism involving both structural and functional components of the airway epithelium [30,31]. This system relies on the synchronised action of ciliated epithelial cells, which generate directional fluid flow through coordinated ciliary beating, and secretory cells that produce the mucus layer containing antimicrobial peptides and surfactant proteins [31,32]. The mucociliary elevator represents a critical first-line defence mechanism, continuously transporting trapped particles, pathogens, and debris from the lower respiratory tract toward the pharynx for elimination [30].

As with all organs and tissues, interaction between each of these cells is likely to be fundamental for the maintenance of airway function and organ homeostasis. Understanding such interactions will be crucial if efforts to tissue engineering human airways are to be successful. Cell–cell interactions may occur via a combination of mechanisms, including secretion of a vast array of functional molecules which can act in a paracrine manner, and mechanobiological events where cell–cell interactions occur via transmission and detection of mechanical changes.

### 2.1. Dynamic Secretome: Mechanical Drivers and Reciprocal Rewiring

The integration of mechanical and biochemical signalling represents a fundamental principle underlying airway function and pathology. Mechanical forces, including cyclic stretch from breathing, matrix stiffness changes during remodelling, and compressive forces from bronchoconstriction, are not merely passive physical phenomena but active regulators of cellular behaviour [33]. These forces are transduced into biochemical signals through specialised mechanosensitive pathways, creating feedback loops where mechanical stimuli influence secretome composition, which in turn can modify tissue mechanical properties and cellular responses.

The dynamic secretome operates through sophisticated reciprocal rewiring mechanisms that enable bidirectional phenotype switching and cellular reprogramming in response to changing mechanical and chemical environments. This reciprocal rewiring represents a fundamental property of airway cell networks where cells can transition between different functional states through mechanotransduction-mediated feedback [34]. Epithelial cells demonstrate remarkable plasticity, undergoing partial epithelial–mesenchymal transition (EMT) in response to matrix stiffness and TGF-β signalling, while retaining the capacity to reverse these changes when mechanical and chemical cues shift toward homeostatic conditions [35]. Fibroblasts exhibit mechanomemory phenomena, where previous mechanical conditioning influences their transcriptional and contractile responses yet demonstrate reversibility when transferred from stiff to soft mechanical environments, indicating that activation states can be dynamically reprogrammed [36]. The reciprocal nature of this rewiring is exemplified by epithelial–mesenchymal communication networks where healthy epithelium suppresses fibroblast activation through BMP signalling, while activated fibroblasts can promote epithelial differentiation through IL-6 and FGF secretion, creating context-dependent cooperative or competitive loops [37,38]. Matrix viscoelasticity emerges as a critical regulator of cellular plasticity, with slower stress relaxation enhancing chromatin accessibility at pluripotency-associated elements and improving cellular reprogramming efficiency, suggesting that mechanical properties can induce profound epigenetic rewiring rather than merely transient phenotype shifts [39]. This reciprocal rewiring framework reveals that airway pathology involves not just dysregulated signalling, but fundamental alterations in cellular plasticity networks that become locked into pathological states through positive feedback loops involving matrix stiffening, pro-fibrotic secretion, and mechanotransduction pathway activation [40].

**Figure 1:**
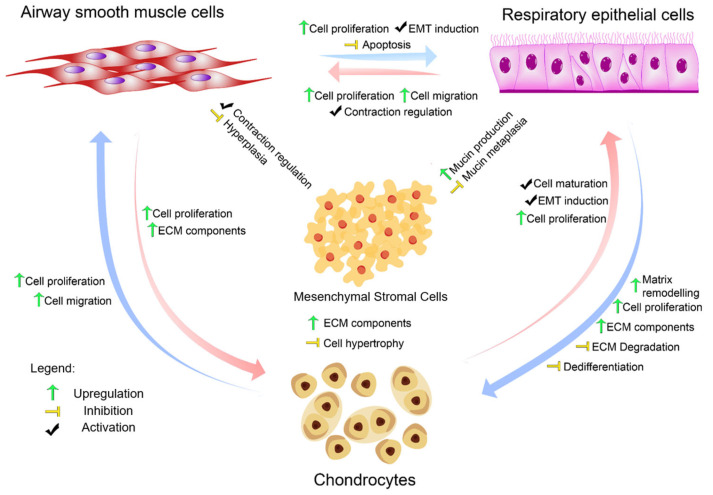
Summary of interaction of airway cells in normal physiology via paracrine signalling, cell–cell interaction, and mechanotransduction cascades. Legend: green upward arrow—upregulation; horizontal flat arrowhead—inhibition; check symbol—enabling function.

A plethora of bioactive components including growth factors and cytokines can be released by the airway cells as described in Table 1, with a summary of interactions in Figure 1. More detailed lists of the secretory profiles of chondrocytes [41,42,43,44,45] MSCs [42,46], ASMCs [47], and AECs [48] can be found elsewhere. Once released, many of these components are capable of autocrine and paracrine effects to induce either local cellular level changes or more wide-spread tissue-level changes in the airway to affect a vast array of signalling pathways that contribute to normal airway function and homeostasis.

Functional molecules released in the airways can be class clustered on their functionality. Mitogenic Growth Factors including Epidermal Growth Factor (EGF), Fibroblast Growth Factor (FGF) and Insulin Growth Factor (IGF) promote cellular proliferation [22,49,50]. Tissue remodelling Growth Factors that modify the expression of extracellular matrix components include Transforming Growth Factor β (TGF-β), Interleukin-1β (IL-1β), vascular epithelial Growth Factor (VEGF), and Connective tissue Growth Factor (CTGF). Each of these factors are involved in wound repair and/or matrix remodelling pathways for the maintenance of tissue homeostasis [51,52,53,54]. Cytokines such as Interleukins act as inflammatory mediators in response to an injurious or inflammatory stimuli or when chronic pathological conditions arise, and they can alter the phenotype and secretions of molecules of airway cells [21,55,56,57,58]. Most of these functional molecules are produced by the cells comprising the airways in either homeostatic and/or pathological processes (see Table 1) and can change the function and/or phenotype of other cells.

An analysis of Table 1 reveals that multiple cell types secrete identical signalling molecules, suggesting both functional redundancy and coordinated responses that ensure robust tissue-level communication [59]. For example, TGF-β is secreted by chondrocytes, ASMCs, and AECs, but in response to different stimuli (IL-1β for chondrocytes, matrix stiffness for ASMCs, and compression for AECs). This distributed secretion pattern ensures robust TGF-β signalling throughout the airway while allowing for stimulus-specific activation patterns that can be fine-tuned by local mechanical and chemical environments.

The mechanotransduction pathways underlying this coordinated secretion involve TRPV4-mediated calcium signalling and YAP/TAZ transcriptional regulation [60]. Recent evidence demonstrates that TRPV4 is necessary for matrix stiffness- and TGF-β1-induced responses across multiple cell types, suggesting a conserved mechanochemical coupling mechanism that enables coordinated secretome regulation (Sharma et al., 2019) [61]. This mechanistic conservation explains how different cell types can produce coordinated responses to mechanical stimuli while maintaining cell-type-specific secretion profiles.

Similarly, VEGF secretion by MSCs (hypoxia-stimulated), chondrocytes (hypoxia-stimulated), ASMCs (injury-stimulated), and AECs (hypoxia-stimulated) creates overlapping angiogenic signals that respond to diverse pathological conditions. The mechanochemical regulation of VEGF secretion has been directly demonstrated in MSCs, where matrix rigidity and cyclic compression increase VEGF secretion via YAP-dependent mechanisms [62]. This redundancy provides resilience against single-cell-type dysfunction while enabling coordinated vascular responses that are essential for tissue repair and remodelling.

The diversity of stimuli listed in Table 1 demonstrates how different cell types serve as specialised sensors for distinct environmental changes, with mechanotransduction pathways providing the molecular machinery for stimulus detection and response coordination. AECs respond to external stimuli (cigarette smoke, compression) through mechanosensitive pathways that couple physical forces to transcriptional programmes (Kilic et al., 2019) [5]. ASMCs respond to mechanical changes (matrix stiffness, stretch) through stiffness-mediated mechanosensation mechanisms that have been characterised on linear stiffness gradient systems [63].

Chondrocytes respond to both mechanical (fluid shear stress) and inflammatory signals (IL-1β) through TRPV4 and Piezo channels that mediate mechanosensing of the biomechanical microenvironment [64]. This dual sensitivity enables chondrocytes to integrate mechanical and inflammatory inputs, creating coordinated responses that address both structural and immunological aspects of tissue remodelling. MSCs respond primarily to tissue damage signals (hypoxia, inflammation) with their secretome profiles being dynamically regulated by mechanical cues through YAP-dependent mechanisms [65].

This distributed sensing network ensures comprehensive environmental monitoring and appropriate tissue-level responses. The specificity of stimulus-response relationships also suggests potential therapeutic windows where targeting specific mechanotransduction pathways could modulate secretion patterns without disrupting normal homeostatic functions [33].

**Table 1:** Secretion of functional molecules of airway cells and their effects.

Secretory Signal	Stimulant	Effect on Airway Cells	References
Secreting Cell: Mesenchymal Stromal Cell			
Activin A		Differentiation regulation	[66,67]
Angiopoietin-1		Vascular stabilisation	[68]
Angiopoietin-2	Shear forces	Angiogenic remodelling	[69]
Bone morphogenic protein-2 and 4	Cyclic tensile strain, PGE2		[70,71,72]
Connective tissue Growth Factor		Fibrotic remodelling	[46,73,74,75]
Fibroblast Growth Factor-2	Hypoxia, TNF-α	Proliferation effects	[76,77]
Hepatocyte Growth Factor	Hypoxia, TNF-α	Epithelial repair	[76,77]
Insulin Growth Factor-1	Hypoxia, TNF-α	Proliferation effects	[76,77,78,79]
Interleukin-1		Inflammatory signalling	[79]
Interleukin-6, 19, 23	Cyclic tensile strain, Dexamethasone	Inflammatory modulation	[80]
Interleukin-7		Lymphocyte support	[81]
Interleukin-8		Neutrophil chemotaxis	[80]
Interleukin-10, 19, 20		Anti-inflammatory effects	
Osteoprotegerin			[67]
Platelet-derived Growth Factor		Proliferation effects, Remodelling signal	[82]
Transforming Growth Factor-β	Hypoxia, TNF-α	Airway cell contraction and ECM remodelling across cell types	[67,77,83]
Vascular endothelial Growth Factor	Hypoxia, TNF-α	Angiogenic/remodelling cues affecting multiple airway cells	[67,76,77,78,79,84]
Secreting cell: Chondrocyte			
Adrenomedullin	Hypoxia	Vasodilation, anti-apoptotic	[85,86]
Angiopoietin-like 4	Hypoxia		[87]
Angiopoietin-like 7	Mechanical stress	Angiogenic signalling	[46,73]
Connective tissue Growth Factor	TGF-β, mechanical stress	Induces EMT in airway epithelial cells; supports perichondrium formation via fibroblast generation	[46,73,74,75,88]
Chitinase 3-like 2	Inflammatory cytokines	ERK activation	[46,73,89]
Epidermal Growth Factor	EGF	Triggers chondrocyte PGE2 release, which increases AEC proliferation	[90,91]
Fibroblast Growth Factor-2	Interleukin-1β	Maintains epithelial integrity; supports barrier/homeostasis	[92]
Interleukin-6	Fluid shear stress	Pro-inflammatory activation	[93]
Nitric Oxide	Interleukin-1	Vasodilation	[94]
Osteomodulin		Regulates mineralization	[46,73,95]
Prostaglandin E2		Increases proliferation of AECs (via paracrine loop).	[90]
Transforming Growth Factor-α			[91]
Vascular endothelial Growth Factor		Angiogenic/remodelling cues affecting multiple airway cells	[96]
Secreting cell: Airway Smooth Muscle Cell			
Adrenomedullin	IL-1β, TNF-α	Vasodilation, angiogenesis	[97,98]
Amphiregulin	TNF-α, IL-4	In AECs: ↑VEGF, ↑PGE2, ↑COX-2, ↑CXCL8; modulates ASM contraction/proliferation	[99,100]
Connective tissue Growth Factor	Injury, TGF-β	Overexpression promotes AEC senescence (pathologic); may drive EMT-like changes	[101,102,103]
Fibroblast Growth Factor-2	TNF-α, IL-1β M	Mitogenic across airway cells (general)	[98,104]
Fibroblast Growth Factor-9	Hypoxia, mechanical stress	Mitogenic across airway cells (general)	[105,106]
Fibroblast Growth Factor-10	Epithelial injury	Promotes epithelial repair	[103,107]
Interleukin-6	TNF-α, epithelial co-culture	Inflammatory response activation	[107,108,109]
Interleukin-8	Interleukin-1β, TNF-α, epithelial injury	Neutrophil chemotaxis	[99,107,110]
Nerve Growth Factor	Interleukin-1β, inflammatory mediators	Neuronal sensitization	[98,111]
Nitric Oxide	Inflammatory cytokines	Smooth muscle relaxation	[106,108]
Prostaglandin E2	Mechanical stress, cytokines	Bronchodilation, anti-inflammatory	[99,106]
Transforming Growth Factor-α	EGF receptor activation	Epithelial proliferation	[91,100]
Transforming Growth Factor-β1	Mechanical injury, hypoxia	Drives ECM remodelling; promotes pathological changes across cell types (context-dependent)	[104,112]
Vascular endothelial Growth Factor	Angiotensin-2, Endothelin-1, TGF-β1, Bradykinin,	IL-4, IL-5, IL-13, PGE2 Angiogenic/remodelling cues affecting multiple airway cells	[113,114]
TGF-β1	Mechanical stress, injury	ECM remodelling; activates epithelial responses (context-dependent)	[102,115]
Stem cell factor	Neutrophil elastase, Increased matrix stiffness	Mast cell activation	[104,116]
Secreting cell: Airway Epithelial Cell			
Adrenomedullin			[117,118]
Amphiregulin	Cigarette smoke		[119]
Angiopoietin			[117]
Chitinase 3-like 1	Viral dsRNA, chitin	Induces IL-8 secretion	[46,89,120,121,122]
Endothelin-1	Compression	ASMC proliferation, contraction	[117,123,124]
Epidermal Growth Factor		Induces chondrocyte PGE2 release → increases AEC proliferation (paracrine loop)	[91,117]
Insulin Growth Factor-1		Mitogenic across airway cells (general)	[117,125]
Interleukin-1β	Thrombin, Trypsin, TNF-α	Stimulates chondrocyte FGF-2 secretion to maintain epithelial integrity	[29]
Interleukin-4, 10, 13, 22		Induces mucus hyperproduction; promotes ciliated differentiation	[126]
Interleukin-6	Thrombin, Trypsin		[29,127]
Interleukin-8	Thrombin, Trypsin, TNF-α		[29,127]
Nitric Oxide		Relaxes ASM by decreasing Ca^2+^ oscillations	[128]
Platelet-derived Growth Factor (PDGF)	Inflammatory cytokines	Smooth muscle proliferation	[129]
Interleukin-11			[29]
Prostaglandin E2	Thrombin, Trypsin	Relaxes ASM (paracrine effect)	[127]
Prostaglandin D2	Allergen exposure, inflammation	Bronchoconstriction	[130]
Transforming Growth Factor-β1, β2	Thrombin, Trypsin, Hypoxia, Amphiregulin	Promotes ECM remodelling; cross-talk with ASM and cartilage	[29,99,117,131]
Tumour necrosis factor α			[29]
Vascular endothelial Growth Factor	Thrombin, Trypsin	Angiogenic/remodelling cues affecting multiple airway cells	[99,127,131]

### 2.2. Mechanotransduction and ECM Feedback: YAP/TAZ Networks Unveiled

The lungs are an inherently mechanical organ, subjected to constant cycles of stretching and relaxation during breathing. An adult on average takes 12–20 breaths per minute during tidal breathing [132]. The cells comprising the airways are therefore adapted to this mechanical environment and in many cases can respond biologically to it in a process known as mechanobiology. In fact, mechanical processes are critical to normal lung development and regrowth of the lung following surgical resections [17]. This is supported by the emerging role of YAP (Yes-associated protein) and TAZ (transcriptional coactivator with PDZ-binding motif) transcription factors as mediators for mechanical stimuli. YAP/TAZ signalling has emerged as a central mechanotransduction pathway in airway cells, with recent studies demonstrating context-dependent roles in epithelial repair versus pathological remodelling. Sustained YAP/TAZ activation promotes aberrant alveolar epithelial cell differentiation and drives persistent fibrotic remodelling, while controlled activation supports regenerative processes [46,133].

YAP/TAZ are main effectors of the Hippo pathway which ultimately affect cell growth, proliferation, and inhibition of apoptosis [134]. Stretching the matrix in which cells are embedded can activate YAP/TAZ, which in turn deactivates contact inhibition and thereby activates proliferation [133]. Furthermore, it has been shown that stretch-mediated activation of TGF-β regulates macrophage function [135], suggesting that breathing may play a crucial role in maintaining immune homeostasis in the lung.

Current single-cell transcriptomics and mechanobiology studies have revealed that airway cells possess a sophisticated mechanosensing apparatus, including Piezo1 channels and YAP/TAZ signalling pathways, that directly couples physical stimuli to cellular responses and paracrine signalling programmes [48,73,117,136]. Piezo1 has been identified as a critical mechanosensor in airway smooth muscle cells, where single-cell hypertrophy promotes contractile function through Piezo1-mediated YAP autoregulation [136]. The RhoA/ROCK pathway functions as a key mechanotransduction cascade in airway remodelling, with enhanced activation observed in asthmatic airways leading to increased smooth muscle contractility and matrix deposition [44].

Dynamic nucleocytoplasmic shuttling of YAP/TAZ in response to tissue stretch is crucial for proper airway branching morphogenesis and alveolar cell differentiation [137]. During lung morphogenesis, nuclear YAP drives actomyosin-mediated tension via RhoA–ROCK signalling, a process required for normal bronchial tree formation [137]. Dysregulation of this YAP/TAZ mechanotransduction axis can lead to branching defects and aberrant cell differentiation, underscoring its importance in lung organogenesis [137].

Airway cells also sense and respond to extracellular matrix (ECM) stiffness via integrin–cytoskeletal pathways. On stiff substrates, YAP/TAZ translocate to the nucleus and actively drive gene transcription, whereas on soft matrices YAP/TAZ remain sequestered in the cytoplasm [33]. This stiffness-dependent YAP/TAZ activation can occur independently of the canonical Hippo kinases, instead relying on cytoskeletal tension; inhibiting Rho/ROCK-mediated actin polymerization prevents YAP/TAZ nuclear accumulation [33]. These findings demonstrate that changes in ECM rigidity are directly translated into altered cell proliferation and differentiation programmes in the lung through mechanotransduction.

Mechanosensitive ion channels further expand the lung’s ability to transduce physical forces. The TRPV4 cation channel, for example, is expressed in airway smooth muscle and epithelial cells and opens in response to mechanical stimuli such as stretch or osmotic swelling [138]. Activation of TRPV4 causes a Ca^2+^ influx that can trigger downstream signalling; in smooth muscle, TRPV4-mediated Ca^2+^ entry contributes to bronchoconstriction and airway remodelling, while in epithelial cells it can influence ciliary beating and cytokine release [138]. Thus, channels like TRPV4 provide a rapid link between mechanical perturbations and cellular responses in the airways.

Mechanical forces can even modulate immune function in the lung. Increasing matrix stiffness has been shown to induce macrophage polarisation toward a pro-fibrotic phenotype via YAP-dependent transcription [33]. Likewise, stiffness-induced YAP activation in the lung microenvironment can suppress T-cell proliferation through metabolic reprogramming [33]. These examples illustrate that an imbalanced mechanical environment can directly skew immune responses, linking mechanobiology to inflammation and tissue remodelling in the lung.

### 2.3. Secretome Mechanotransduction Feedback Signalling: Closed Loop Model and Control Points

There is a crosstalk between mechanobiology and secretory pathways in the lung. Mechanical signals can influence the secretion of functional molecules in the airway, which can then contribute to a positive feedback manner to cellular and tissue-level mechanical action such as contraction and relaxation. For example, mechanical signals including increased stiffness, stretching, and contraction can cause the activation and release of TGFb from epithelial cells and ASM cells [139,140,141]. Once activated, TGFb has the potential to influence the contraction of airway cells [142,143] as well as regulate remodelling processes [144,145,146].

**Figure 2:**
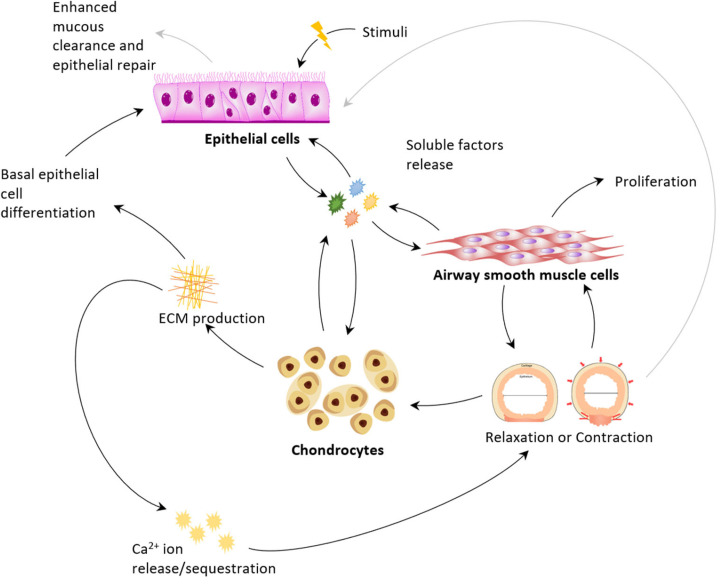
General mechanotransduction dynamics in the airways. Epithelium-induced ASM contraction coming from external stimuli such as particulates from inspired air or epithelial damage cascades throughout the airways by release of functional molecules that act as a stimulant to the ASMCs. Cyclic contraction or relaxation of ASM due to epithelial-derived agonists affects the whole organ in which a compressive force is induced, initiating ECM production in chondrocytes that both serves as a Ca^2+^ ion reservoir for modulation of ASM activity and as a scaffold on basal epithelial cells on the interface of cartilage and epithelium, where epithelial cells differentiate further into a pseudostratified epithelium with diverse phenotypes. Cyclic compressive force also increases ciliary beat frequency of AECs and ATP release, enhancing mucous clearance and inducing epithelial repair.

Altered mechanical movements can then drive cells to secrete growth factors and cytokines and so perpetuate the loop. Figure 2 shows the proposed feedback loop on secretome–mechanotransduction pathways, where cellular-to-organ-level responses are mapped with corresponding secretome and mechanobiological actions. Evidence of the feedback mechanism can be observed in the early works of Fedan’s team, where it has been shown ex vivo that the presence of cartilage and epithelium affected the contraction of the ASM [147,148]. Removal or denudation of cartilage and epithelium lead to the altered sensitivity of airway smooth muscle.

Airway epithelial cells sense compressive or shear stress and activate mechanotransduction cascades that change gene expression and drive release of paracrine mediators relevant to airway tone and remodelling. Several recent studies show mechanical compression/stretch elicits Ca^2+^-dependent signalling and secretion of ATP, prostaglandins, and extracellular-matrix proteins/vesicles that can influence neighbouring cells and tissue state. Compression of primary human bronchial epithelium alone produces inflammatory, repair, and fibrotic transcriptional programmes that mirror asthmatic signatures, consistent with force-driven epithelial activation by bronchospasm [149]. Mechanical compression of differentiated human bronchial epithelial cells increases tenascin-C expression and secretion in extracellular vesicles, implicating epithelial mechano-response in ECM remodelling [150]. TRPV2 in primary bronchial epithelial cells mediates mechanically induced ATP release, demonstrating a channel-dependent epithelial nucleotide signal triggered by mechanical stress [151]. Acetylcholine stimulated prostaglandin E2 release from tracheal epithelium and thereby induced smooth muscle relaxation in rat tracheal rings, showing an epithelial-derived biochemical pathway that changes ASM contractility [152]. Recent reviews highlight store-operated Ca^2+^ entry and other Ca^2+^ pathways as central hubs by which epithelial mechanostimuli regulate mediator production and secretion [153].

Airway smooth muscle functions as the downstream effector that detects mechanical and biochemical inputs and converts them into contractile or relaxant responses; recent work describes specific mechanosensors and Ca^2+^ regulators in ASM. The cited studies identify stretch-responsive pathways, Piezo-mediated responses, and stiffness-sensing that alter ASM biomechanics and contractility. STIM1 mediates stretch-induced signalling in human ASM, upregulating mechanosensitive channels (Piezo1/2), Orai1, and inflammasome components and modifying Ca^2+^ responses relevant to contractility and remodelling [154]. Chemical activation of Piezo1 (Yoda1) in cultured ASMCs produces transient Ca^2+^ signals and long-term reductions in cell stiffness and traction force consistent with pro-relaxation biomechanical changes [155]. Human ASM cells show stiffness-dependent changes in cell size, α-SMA expression, YAP translocation, and other mechanotransduction readouts, linking ECM mechanics to ASM contractile phenotype [63]. Comprehensive reviews place TRP and Piezo families at the centre of lung mechanosensing, influencing ASM behaviour and organ-level responses [156].

Recent reviews and syntheses frame epithelial–ASM mechanochemical interactions and ECM mechanotransduction as integrated regulatory pathways that maintain (or disrupt) airway homeostasis; these works link cellular mechanosensing to tissue remodelling and organ function. The literature emphasises multiscale mechanotransduction and ECM feedback as drivers of pulmonary pathophysiology and as potential therapeutic targets. Mechanotransduction coupled to extracellular matrix composition and mechanics is highlighted as a key driver of lung pathologies and of drug responsiveness, connecting cellular mechanosensors to organ-level homeostasis and disease progression [157]. Reviews of mechanosensitive channels in lung health discuss how epithelial and ASM channels (TRP, Piezo) mediate force to signal transduction that coordinates mucus hydration, inflammation, remodelling, and contractile behaviour, supporting a systems-level regulatory role [156]. Experimental demonstrations that epithelial compression induces asthma-like signatures and releases paracrine mediators (ATP, PGE2, ECM/EVs) provide mechanistic nodes that feed into ASM and ECM responses implicated in sustained changes to airway function [149,150,151,152].

The importance of such cellular interactions has been demonstrated in vivo. Mouse mutants genetically manipulated to inhibit cartilage or smooth muscle formation in the developing airways have shown the co-dependence of cartilage and smooth muscle upon growth, the important role of the cartilage on the epithelium differentiation, and that the absence of either tissue resulted in malformation of the airway [158]. This study highlights the necessity of understanding the interplay between cell types in the airway, and so we shall then look closely at the cellular level interactions that drives these processes altogether.

## 3. Interactions of Airway Cells

### 3.1. Physiology: Epithelium as Activator Smooth Muscle as Actuator Cartilage as Regulator

The cellular niche of the airways provides pathways for the homeostasis of the surrounding tissues. Structurally, the lumen of the airway is covered by AECs that are supported by the cartilage and modulated by ASMCs [19]. In the physiological condition, it can be hypothesised that the AECs receive stimuli from the environment and provide cues for airway dynamics; cell- and tissue-level event cascades initiated by the secretome can drive multiple responses in the airways (Figure 2). Growth factors secreted by AECs [48,119], ASMCs [101,102,105], and chondrocytes [46,73,89,120] can act as mitogens across all cell types, thereby driving cellular proliferation [22,159,160], migration [161], and cell survival through protease-activated receptor-2–, AKT-, ERK-, and p38-MAPK-dependent pathways [22,162,163]. Several of the factors released are induced either by other growth factors or via mechanotransduction, prompting a cascade of events to maintain cellular homeostasis (see Table 1 for a list of factors and known stimulants).

Apart from cellular proliferation, the integrity of the airways is also dependent on the secretion of molecules and proteins for maintenance of the extracellular environment.

Recent studies have identified specific molecular pathways mediating epithelial–smooth muscle communication. Airway epithelial cells promote smooth muscle cell proliferation by activating the Wnt/β-catenin pathway, while semaphorin3E/PlexinD1 signalling represents a novel regulatory axis in COPD pathogenesis [139,141]. These interactions are dynamically regulated by mechanical forces and inflammatory stimuli.

Acting as the mechanical framework of the airways, chondrocytes produce mechanically resilient ECM components like collagen type II and proteoglycans that are necessary for the load-bearing capacity of the airway [25,26]. These components can be upregulated through (1) the secretome, by factors secreted by AECs [29,48,123,125], and (2) mechanotransduction, by the contraction/relaxation mechanism of ASMCs that relates to TGFb activation [164]. The latter mechanism might be explained by the fact that chondrocytes, when subjected to cyclic compressive stress, upregulate their ECM production [165,166]. This suggests that the mechanotransducive events produced by the ASMCs can influence chondrocyte functionality. In the remodelling phase, AECs secrete several cytokines that may aid chondrocytes in their growth phase, inducing release of matrix remodelling enzymes for tissue expansion [51,52,53]. Additionally, a paracrine feedback response of EGF from AECs to chondrocytes can trigger release of PGE_2_ [90], which increases the rate of proliferation of AECs through induction of c-Jun and three-phosphoinositide dependent protein kinase-1 (PDK1) pathways [167].

Chondrocytes in turn regulate the physiological responses of AECs and ASMCs. The maintenance of epithelial integrity is dependent upon the Fibroblast Growth Factor 2 (FGF-2) [168], which chondrocytes secrete in response to Interleukin-1β (IL-1β) [92]. IL-1β is produced by AECs after an insult from an allergen or related stimuli [29]. This potential feedback loop provides an example of AECs responding to an environmental insult via secretion of mediators leading to activation of nearby chondrocytes to maintain AEC and airway homeostasis. In terms of ASMC regulation, ECM components produced by chondrocytes can serve as a reservoir of Ca^2+^ ions due to their highly negative net charge. This modulates ASM contraction [147,169], which affects its differentiation and growth. The feedback loop between the production of cartilage ECM and ASM contraction/relaxation affects the extent to which the mechanical effects (contraction and relaxation) of the airway is induced. Such effects have been investigated by Ramchandani et al. [170], where the proportion of cartilage to ASM influences the mechanical compliance of the airways. These interaction varies highly from the developmental to maturation state of the airways [170].

Advanced iPSC-derived multi-cellular co-culture systems have enabled detailed investigation of airway barrier integrity and intercellular signalling. These models incorporate epithelial, mesenchymal, endothelial, and immune cell interactions, providing physiologically relevant platforms to study disease mechanisms and therapeutic interventions [158]. Human and mouse pluripotent stem cell platforms now combine differentiated epithelial, mesenchymal, endothelial, and immune lineages to recapitulate airway cellular neighbourhoods and study interlineage signalling in vitro. These systems range from multi-lineage organoids to air–liquid interface (ALI) co-cultures that permit both paracrine and contact-dependent crosstalk and functional readouts of barrier, differentiation, and progenitor behaviour.

Multi-lineage ALI iAirway assembles iPSC-derived epithelium, mesenchyme (epithelial–mesenchymal organoid cores), endothelium, and macrophages in an ALI format to study barrier responses and pathogen/toxin effects while preserving cross-talk between compartments [158]. Mouse iPSC-derived lung-specific mesenchyme (iLM) can be combined with engineered epithelial progenitors to self-organise into 3D organoids with juxtaposed epithelium and mesenchyme; co-culture increases epithelial progenitor yield and modifies epithelial and mesenchymal differentiation programmes, showing functional reciprocity [171]. Human ESC/iPSC protocols generate airway organoids containing epithelial and mesenchymal populations and can be invested with mesodermal derivatives, enabling modelling of chondrogenesis, smooth muscle formation, and epithelial maturation within one system [172]. Co-culture experiments demonstrate that airway epithelium actively drives airway smooth muscle (ASM) phenotype switching (proliferative/pro-inflammatory) via secreted factors and microRNA-dependent pathways, emphasising the value of paired cultures to study mesenchymal responses [107].

iPSC co-cultures both exploit and reveal the same morphogen axes that pattern the embryonic airway; canonical Wnt, BMP, FGF, and Shh pathways act in reciprocal, context-dependent ways between the epithelium and mesenchyme. In vitro modulation of these signals in mixed-lineage cultures recapitulates lineage choices (chondrocyte vs. smooth muscle), regional identity, and differentiation timing seen in embryos. Bidirectional Wnt signalling between the endoderm (epithelium) and mesoderm (mesenchyme) is necessary to induce mesenchymal tracheal identity (Tbx4) and to generate periodic cartilage and smooth muscle structures in ESC/iPSC-derived cultures; human LPM requires WNT but needs SHH coactivation for correct tracheal mesoderm specification [173]. BMP4 and WNT cooperate during tracheal mesenchyme morphogenesis; mesenchymal BMP4 promotes chondrogenesis and restrains trachealis muscle, and loss of mesenchymal BMP perturbs Wnt target expression and lineage outcomes, findings that are recapitulated in co-culture and organoid assays [174]. Mesenchymal BMPR1A–BMP signalling promotes airway SMC differentiation via Smad-independent pathways (p38 MAPK) rather than solely by canonical Smad1/5, linking receptor-level BMP input in mesenchyme to SMC gene programmes in vitro and in vivo models that inform iPSC differentiation strategies [175]. Standard iPSC airway protocols use FGFs to drive epithelial and mesenchymal maturation and require retinoic acid/SHH (depending on species and protocol) to pattern ventral foregut and lateral-plate mesoderm derivatives, so co-cultures exploit timed FGF/SHH application to produce chondrocytes, smooth muscle, and mature epithelium [172,173].

Overall, a broad and complex interaction is seen between cell types. Fine-tuning of secretory and mechanotransducive effects initiated by resident cells in the airways are necessary for homeostasis conditions, and these conditions arise as concerns in the equilibrium achieved in the developing airway.

### 3.2. Development: ASM FGF10 and Peristalsis Pattern Cartilage and Epithelial Differentiation

The developmental aspect of airway morphogenesis represents a critical framework for understanding how mechanotransduction and secretome interactions establish the foundational architecture of respiratory tissues. During embryonic development, coordinated cellular interactions between airway smooth muscle cells (ASMCs), epithelial cells, and chondrocytes create the structural and functional blueprint that defines adult airway homeostasis [176,177]. This developmental paradigm is particularly relevant because pathological airway conditions often recapitulate these embryonic signalling pathways, suggesting that understanding developmental mechanobiology provides therapeutic insights for airway diseases. The temporal orchestration of mechanical forces, growth factor gradients, and cellular differentiation during development establishes the bidirectional feedback loops between secretome-mediated signalling and mechanotransduction that persist throughout adult life [158,171,177].

**Figure 3:**
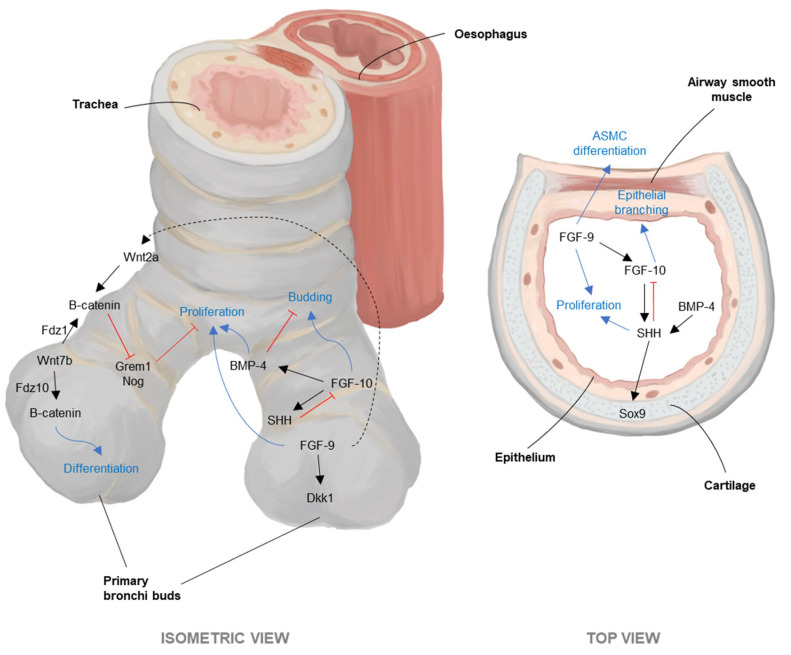
Signalling pathways in the developing airways. Key functional molecules are Sox9 (cartilage formation), FGF-10 (epithelial branching), and FGF-9 (ASMC differentiation).

The overview of human airway development is outlined by Pansky; starting at week 4 of embryonic life, a laryngotracheal groove forms and deepens. This part forms the primitive airways, with two bronchi buds forming at week 5 [178]. Differentiation of ASMCs starts at the end of week 7, and, a week after, the visibility of the tracheal cartilage through mesenchymal rudiments can already be seen. The tracheal cartilaginous mass further develops in a cranial to caudal fashion, occurring concurrently as fibroelastic tissues between the rings and airway smooth muscle within the c-ring; a cartilage gap arises within two weeks. On the other hand, cartilage development is seen at week 10 in primary bronchi and week 12 in segmental bronchi [178]. Additionally, ASMCs aid the posterior wall formation of the larger bronchi where cartilage tissue is absent. In the trachea, ciliated epithelium appears at week 10, and mucosal glands are seen from week 12, similarly following a craniocaudal direction [176]. The bronchi, however, form a ciliated epithelium at week 12 and a week afterwards produce mucous glands [178]. At week 20, the microscopic features of the trachea are visible, followed by the bronchus; both the final forms are reached post-natal stage [176]. A general scheme of developmental signalling is shown in Figure 3.

The importance of mesenchymal cells on the developing organ highlights the potentially important role of airway smooth muscle for maturation of the airway, as it is differentiated earlier than the rest of the cells. ASMCs drive the layout structure of the airways via FGF-10 [177] signalling, which is required for lung morphogenesis [179]. Airway ASM peristalsis in the prenatal environment [180], which increases frequency towards birth [181], cues intermittent c-ring structure formation in the trachea as mechanical contraction varies periodically across the tissue. Cartilage maturation can also be observed in co-culture of chondrocytes with ASMCs, increasing production of collagen II and IX by chondrocytes and increasing their pro-chondrogenic activity [182]. Both cell types regulate each other on proliferation, differentiation, and airway biomechanics [158], which in turn affect the epithelial coverage and differentiation of the airways [183].

As the ASM forms, it pushes the luminal fluid to the terminal ends of the airways by peristaltic phasic contraction [184]. Rhythmic ASMC contractility is observed due to intercellular calcium wave propagation [185]. These calcium waves can then affect calcium-activated chloride channels that release chlorine ions to the adjacent AECs, stimulating the secretion of prenatal lung liquid. This is known as the fluid pump hypothesis [186], where the interaction between ASMCs and AECs via mechanotransduction and secretome pathways are highlighted as necessary to support lung development. AECs can also release nitric oxide (NO), which can act as an ASM relaxant by decreasing the Ca^2+^ oscillation on cells [187]. NO is found to be released in the bronchial and proximal bronchiolar epithelia in the foetal state, suggesting its contribution to airway morphogenesis [188]. Phenotypically, ASMCs that are in a non-contractile state are in a proliferative state, with the associated reduced expression of contractile components such as smooth muscle myosin heavy chain (sm-MHC), calponin, sm-alpha-actin, and desmin [189]. A similar effect on ASMC relaxation can also be induced by PGE_2_ [190], which can be secreted from AECs [191]. It is therefore apparent that the airway epithelium can regulate contraction of ASM [191,192,193] in the early lung development. Since the epithelium is the exposed layer of the airways, stimuli coming from external sources can cause the release of agonists that regulate the contraction and relaxation of ASM. AECs can be seen to provide biochemical stimuli, and ASMCs can be seen to provide mechanical stimuli.

Cartilage, on the other hand, can influence the phenotype and/or function of AECs. The absence of cartilage in the trachea has been proven to decrease basal cell density, precocious development of club cells, and the KRT14^+^ cell population (a cell that has high reparative effects on the airways), all likely due to altered FGF signalling [158]. Chondrocytes express and release of Connective Tissue Growth Factor (CTGF) [46,73,74,75,88], which can induce epithelial–mesenchymal transition (EMT) [194], generating fibroblasts [195] between the layers of where the AEC and chondrocyte reside. Such fibroblasts can proliferate under the presence of FGF [196] (which chondrocytes also produce), and so in time develop a perichondrium layer. The presence of a perichondrium in the intersection of epithelial and cartilage tissue layers could, therefore, result from the paracrine effects of the chondrocytes on the basal layer of the respiratory epithelium.

Critically, these developmental signalling pathways depicted in Figure 3 are reactivated during airway regeneration following injury, demonstrating that embryonic patterning programmes serve as templates for adult tissue repair [197,198]. Post-injury regeneration recapitulates key elements of developmental morphogenesis, where Wnt signalling activation promotes basal cell proliferation and differentiation, while BMP pathway modulation controls the balance between proliferation and differentiation phases essential for proper epithelial restoration [199,200]. FGF-10 signalling, crucial for embryonic epithelial branching, becomes reactivated after airway injury to stimulate basal stem cell responses and coordinate epithelial repair [201,202]. ASMCs, upon onset of injury, express FGF-10 [177], which promotes epithelial repair [103]. Similarly, Sox9-mediated pathways are reinitiated during injury repair to restore structural integrity, while maintaining progenitor cell states essential for airway regeneration [203,204]. Results from McVicar et al. also reinforce the likelihood of the cartilage as a developmental niche for airway basal cells [158], supporting the epithelial regeneration pathway. The temporal dynamics of these reactivated developmental cascades determine successful regeneration outcomes; early Wnt activation drives proliferative expansion, followed by precise signalling restoration to promote proper differentiation [197,205]. The crosstalk between cells helps maintain the integrity of the airways, following the same developmental pathways for whole-organ homeostasis. Understanding this developmental/regenerative pathway overlap is essential for designing therapeutic interventions that harness endogenous repair mechanisms while preventing pathological pathway dysregulation that can lead to aberrant remodelling. However, in pathological conditions, signalling and biomechanics are compromised, resulting in unsynchronised overgrowth or degradation of tissues within the airways, coupled with overlapping signals that perpetuate the condition.

Further elucidation of the developmental pathways via multicellular iPSC systems operationalizes fundamental embryology—reciprocal epithelial–mesenchymal signalling, spatially patterned progenitor competence, and mechanical/contractile feedback; these have been reproduced and are experimentally tractable in vitro. Recapitulation both validates embryologic models and allows researchers to perturb single axes while preserving the multicellular context. Co-culture organoids and ALI platforms model the epithelial–mesenchymal trophic unit (EMTU), showing how epithelial signals instruct mesenchymal differentiation, and, conversely, mesenchyme alters epithelial progenitor kinetics and fate—paralleling concepts from developmental lung biology and asthma co-culture studies [171,206].

Single-cell and live-imaging studies show that the sub-epithelial mesenchyme gives rise to airway smooth muscle and that Wnt activation induces early cytoskeletal (F-actin) and adhesion programmes that change epithelial morphology; iPSC co-cultures reproduce these spatiotemporal cues, allowing for the dissection of biochemical versus mechanical contributions to morphogenesis [207]. By rebuilding developmentally patterned signalling (Wnt↔BMP↔FGF↔Shh) in controlled multicellular cultures, iPSC co-cultures link embryologic mechanisms to human-specific differentiation outcomes and disease modelling (e.g., congenital airway malformations, ASM remodelling), and provide platforms to test how altering one compartment shifts the developmental trajectory of its neighbours [173,174,175].

### 3.3. Disease: Developmental Programmes Misapplied in Asthma and COPD and the Feed-Forward Stiffness Trap

The mechanobiology and signalling pathways in the airways are significantly altered in pathological conditions. Indeed, accumulating evidence indicates that aberrant mechanotransduction is a unifying feature of chronic airway diseases. In asthma and COPD, aberrant mechanobiology drives pathological remodelling through multiple interconnected pathways. Dynamic mechanical stimulation studies using advanced biomaterial systems demonstrate that cyclic strain induces mucus hypersecretion in human bronchial organoids, while altered matrix mechanics promotes fibroblast activation and collagen deposition [208,209]. Another example is the persistently elevated YAP/TAZ signalling observed in the fibrotic airway remodelling of asthma and COPD, analogous to its pro-fibrotic role in idiopathic pulmonary fibrosis [210]. Such dysregulated mechanical signalling can drive excessive cell proliferation and matrix deposition, suggesting that pathways maintaining normal airway structure become pathologically overactive in disease.

**Figure 4:**
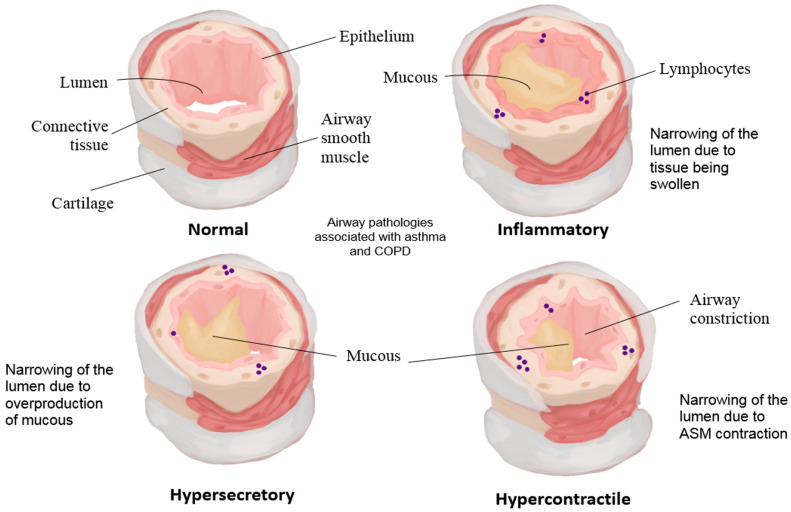
Anatomy of pathologies in bronchus. Inflammation, mucous overexcretion, and hypercontractility of the ASM are few of the pathologies seen in the trachea and bronchi.

Current information on murine and porcine cystic fibrosis models show cartilage and ASM abnormalities prior to subsequent epithelial defects [211,212,213]. Similarly, in the developing airways, epithelial defects are also seen when there are aberrations in cartilage and ASM formation [158]. Mechanistic parallels between lung development and adult disease are now being recognised. The same Hippo–YAP/TAZ pathway that is crucial for normal airway morphogenesis can be inappropriately reactivated in asthma, promoting abnormal cell growth and airway remodelling. In other words, developmental mechanotransducive signals, when dysregulated, may contribute to the cascade of changes seen in the asthmatic airway. The hypothesis on recapitulation of developmental signalling in the adult state could also be reflected in the pathogenesis in asthma—inferior mechanical properties of the cartilage [214], increased ASM mass [215,216] coupled with hypercontractility [217,218], and an altered AEC phenotype whose integrity is lost [219]. Figure 4 shows the anatomy of COPD and asthma in the lower airways.

As previously discussed, mechanical events such as the cycles of contraction and relaxation in the airways can affect the release of soluble factors, which in turn can have paracrine effects of mediator release and mechanobiology. However, excessive mechanical activity and elevated soluble factors release can arise from pathologic conditions, such as asthma, which shifts the equilibrium so that cellular responses are aberrant. In addition, acute mechanical injury to the epithelium can initiate inflammatory cascades that exacerbate airway disease. It was recently shown that bronchiolar club cells act as mechanical damage sensors via the TRPV4 channel; when epithelial junctions are compromised, TRPV4-mediated Ca^2+^ signalling in club cells triggers release of “danger” signals that drive type 2 inflammation and allergic sensitization [220]. This mechanosensory pathway directly links epithelial barrier stress to asthma pathogenesis, providing a molecular explanation for how repeated epithelial damage (even in the absence of infection) can lead to chronic inflammation. These events alter the microenvironment, specifically the ECM components, and can lead to the perpetuation of pathologic conditions in the airways. For example in asthma, airway remodelling is vividly seen; ASM mass is increased [216], becoming hypercontractile [217], and degradation of cartilage with increased perichondral fibrosis occurs [214].

Excessive mechanical stress on the asthmatic airway wall can itself exacerbate pathology. A recent study demonstrated that increasing the ECM stiffness in airways (by exogenous collagen cross-linking) is sufficient to provoke excessive airway narrowing, even in the absence of inflammation [33]. This finding suggests that a stiffer airway matrix—a hallmark of asthmatic remodelling—directly heightens bronchial hyperreactivity. In asthma, such feed-forward mechanobiological disruption can create a vicious cycle in which remodelling begets stiffness, and stiffness in turn triggers hyperconstriction and further remodelling. This is affected by altered crosstalk between ASMCs, AECs, and chondrocytes. Asthmatic ASMCs release increased amounts of PGE_2_ [21] that can cause delayed development of chondrocytes by the inhibition of BMP signalling [221]. Dedifferentiated chondrocytes express elevated levels of smooth muscle actin [222], synthesise collagen type I [223], and acquire a contractile phenotype [222,224]. Additionally, IL-1β, which is involved in the pathogenesis of asthma, can causes FGF-2 release from chondrocytes [92], which could promote fibroblast proliferation in the perichondrium and contribute to perichondral fibrosis in asthma [214]. Furthermore, since ASMC contraction can activate TGF-β [139], which is a protein crucial for ECM production and the pathological remodelling of airways, it is possible that enhanced airway contraction in asthma drives structural changes within the airways, as shown in our previous study [164] Aberrant mechanotransduction in ASMCs is a major driver of asthma’s pathological airway remodelling. Asthmatic ASMCs show abnormally high nuclear YAP levels, which promote excessive proliferation and a hypercontractile phenotype [225]. Moreover, stiffened peribronchial matrices in asthma can further amplify ASMC dysfunction; the matrix protein Fibulin-5, for instance, engages β1-integrins on ASMCs and activates YAP/FOXM1 signalling, enhancing smooth muscle migration and contractility [225]. This YAP-mediated feed-forward loop in ASM cells contributes to sustained airway narrowing and increased bronchial wall thickness in asthma.

The collateral damage brought by structural and morphological changes in ASMCs and chondrocytes relays to the airway epithelium. Amphiregulin, which is highly secreted from asthmatic ASMCs, causes elevated expression of VEGF, PGE_2_, Cyclooxygenase-2, and CXCL8 in AECs [99,226], which can modulate ASMC contraction and proliferation. In addition, the mechanotransducive effect of the ASMCs can also be perpetuated by the secretion of Endothelin-1 by AECs upon compression via ASM contraction to drive further ASMC proliferation and contraction [124]. In chronic pathological conditions, the airway epithelium is dysfunctional [227], and one explanation suggests that this is possibly due to the CTGF release of ASMCs from TGF-β1 stimulation [102] through AEC secretion [29]. It was mentioned in a previous discussion concerning AEC–chondrocyte interaction that CTGF may play a role in the epithelial–mesenchymal transition of AECs, which could also be the case in ASMC-AEC crosstalk. However, in pathologic conditions such as COPD and asthma, CTGF overexpression promotes AEC senescence [228]. These senescent AECs alter the phenotype of the respiratory epithelium in pathological conditions. Peribronchial fibrosis, submucosal gland hypertrophy, and mucous metaplasia are the changes observed in the epithelium [214,229]. There is also growing appreciation for epithelial mechanobiology in chronic airway disease. An asthmatic bronchial epithelium often exhibits loss of integrity and stress-induced changes; studies have found regions of epithelial denudation, reduced ciliated cell numbers, and elevated EGFR and TGF-β signalling even in mild or early asthma, changes observed even in the absence of heavy inflammation [230].

These findings imply that mechanical injury (from repeated bronchoconstriction, coughing, or particulate exposure) contributes to epithelial damage and dysregulation in asthma. Notably, YAP/TAZ, which in healthy airways help restrain goblet cell hyperplasia, become mislocalised or inactivated in injured epithelium—leading to excessive goblet cell differentiation and mucus overproduction [231]. Thus, mechanical stress and YAP/TAZ dysfunction in the airway epithelium together drive the mucous metaplasia and barrier impairment characteristic of chronic asthma. Additionally, since ASMCs are highly proliferative and contractile in these conditions, they can likely affect neighbouring cells due to changes in secretory and mechanobiological processes. Exacerbated by the denudation of cartilage in several pathological conditions, renewal of epithelial integrity is difficult without any external intervention. Smooth muscle and cartilage dysfunction in chronic airway diseases are intimately connected. In both asthma and COPD, bronchial cartilage shows evidence of degeneration (loss of cartilage matrix and viable chondrocytes) along with increased perichondrial fibrosis [214]. This degraded cartilage provides less mechanical support to the airway, making the airway wall more susceptible to narrowing or collapse when surrounding smooth muscle contracts. In emphysema-associated COPD, the problem is compounded by the destruction of alveolar attachments (the elastic fibres tethering small airways open), which removes critical radial tension on the airway walls [225]. The net result of these structural changes is an airway that is abnormally prone to deformation—stiff in some regions and collapsible in others—leading to airflow limitation that is refractory to normal reversal mechanisms.

Organoid and co-culture platforms have been applied to airway disease modelling (fibrosis, asthma, infection), revealing how epithelial injury or inflammatory signalling remodels mesenchyme and how mesenchymal signalling reshapes epithelial fate and ECM, with mechanotransduction and cytoskeletal forces modulating these outcomes [158,232]. In disease-focused co-cultures and EMTU models, epithelial-derived cytokines and growth factors drive ASM phenotype changes toward pro-proliferative and pro-inflammatory states (increased IL-6/IL-8, miR-210, AKT activation), establishing a paracrine axis by which damaged or asthmatic epithelium amplifies mesenchymal remodelling [107,206]. Live-imaging and single-cell studies further show that Wnt-driven mesenchymal patterning coordinates cortical tension and migratory behaviours that influence epithelial morphology, implying mechanotransductive feedback during both normal branching and pathological remodelling [175].

Conditioned media and co-culture experiments show epithelial release of mitogens/cytokines that activate ASMC proliferation, inflammatory gene programmes, and miRNA-mediated repression of tumour suppressors, shifting ASM toward a synthetic/proliferative phenotype relevant to asthma and remodelling [107,206]. Disrupting BMP signalling in mesenchyme alters the cartilage/SMC balance, impairs elastin deposition, and produces cystic or fibrotic airway phenotypes; BMP–WNT reciprocity governs mesenchymal differentiation and thus affects epithelial architecture in disease models [178,179]. Early [174,175] Wnt activation in mesenchyme induces local F-actin accumulation and patterned cortical forces that change epithelial morphology; these cytoskeletal/mechanical cues are accessible in organoid/co-culture systems and likely contribute to disease-associated remodelling when signalling is aberrant [174,175]. Multi-lineage iPSC co-cultures (epithelium + mesenchyme + endothelium + immune cells) recapitulate barrier loss and inflammatory remodelling in response to viral infection or toxins and provide a testbed to probe epithelial–mesenchymal–chondrocyte interactions under pathological perturbation [158,232]. Collectively, recent iPSC-based organoids and layered co-cultures permit the manipulation of Wnt, BMP, FGF, and Shh inputs and allow paired biochemical and biophysical readouts (cytokines, miRNAs, ECM, cortical tension) to dissect epithelial–SMC–chondrocyte crosstalk in development and disease [173,174,175,206].

### 3.4. Mesenchymal Stromal Cells as Global Mediators

There have been several attempts to regulate the pathologies in the airways, from pharmaceutical to cellular-based methods. Looking back at the developmental origins of airway growth, progenitor cells may be the key to recapitulate the regenerative capacity of the airways. Lung mesenchymal stromal cells have been recently receiving attention regarding the possibility for them to replace dysfunctional cells and ameliorate symptoms associated with the pathologies described above.

With the plethora of growth factors, cytokines, and other functional molecules that they produce, MSCs provide many of the necessary conditions for proliferation and phenotype maintenance of other airway cells and therefore interact with each of the airway cell types. Moreover, MSCs are also capable of differentiating into a specific cell type to regenerate the tissue during repair [83]. It has been well documented that MSCs are present in the lungs [233,234] and are capable of differentiating to all classes of airway cells [235,236]. These lung-resident MSCs also contribute significantly to the immune regulation of the airways and are seen vividly in lung allografts where they intervene T-cell expansion [237]. Airway regeneration is mediated by MSCs from cellular and tissue-level damages, where MSC mobilisation is induced by FGF-10 [238] that is secreted by ASMCs in response to injury [103].

Cellular interactions of MSCs and airway cells are best viewed in co-culture systems. Le Visage et al. performed an air–liquid interface transwell co-culture of MSCs and AECs and found that the co-culture retained the capacity of AECs to produce mucins in contrast to AECs alone [239]. This functional retention can be attributed to MSCs’ ability not only to transfer molecules via paracrine secretion but also organelles via tunnelling nanotubes [240]. MSCs’ capability to transfer mitochondria to AECs proved to be beneficial in repairing damage induced by cigarette smoke in rats [240]. Furthermore, MSCs can replace lost AECs in the airway epithelium by differentiating into AECs themselves [241].

Modulation of MSCs in the asthmatic activity of ASMCs is evident in the study of Urbanek’s group. In a murine model, delivery of MSCs in trachea reduced the hyperplastic phase of ASM and lowered the mucous metaplasia of AECs. Also, MSCs decreased the levels of cytokines IL-4, 5, and 13 (these cytokines are involved in the recruitment of immune cells), and upregulated its expression of IL-10 and indoleamine 2,3-dioxygenase [242] (which reduces the rate of proliferation of ASMCs) [243]. This function of MSCs suggests they can modulate the cell proliferation of ASMCs and counteract the pathological condition by regulating present ASMC populations. Moreover, MSCs are likely to differentiate in case of cellular loss for replacement, as in cases of muscular atrophy treated with MSCs. Also, the ASMC contraction/relaxation phase can also be regulated by MSCs [244] due to their capability to transfer mitochondria and Ca^2+^ through tunnelling nanotubes [83], similar to the earlier discussed processes of MSC-AEC interaction. The presence of MSCs in the airways may prevent stenosis by controlling the hypertrophic growth of cells and modulating the contraction induced by ASM.

Co-culture experiments have also highlighted positive reinforcement between MSCs and chondrocytes. Several studies proved that the presence of MSCs in chondrocyte culture increased the expression of glycosaminoglycans and total collagen and eliminated the hypertrophic characteristics of cartilage by reduction in collagen X and MMP 13 expression and the absence of calcification [245,246]. Hypertrophy reduction in both cell types is believed to be the effect of parathyroid hormone-related protein chondrocyte secretion in the presence of MSCs that inhibit alkaline phosphatase and diminish cell enlargement [221,247,248]. Chondrocytes also improve the chondrogenic differentiation of MSCs [249], and, in turn, MSCs promotes proliferation and help maintain the phenotype and microenvironment of chondrocytes by upregulating the ECM protein released in cartilage tissues [250,251]. The mutualism exhibited by cell–cell interaction and paracrine effects of MSCs and chondrocytes is crucial in maintaining the functionality of cartilage in the airways to preserve the structural integrity of the whole organ. Overall, MSCs possess a regenerative capability, as well as modulate important functions of each cell type in the airway by secreting necessary growth factors and cytokines, in addition to transferring organelles such as mitochondria via tunnelling nanotubes.

As the predicted gold standard of airway management is tissue engineering, understanding pathways of the homeostasis maintenance of the airways could potentially unlock successful graft generation. Each cell within the airway secretes its set of functional substances, affecting local and global scales in the airway. An organ-scale effect is induced via mechanotransduction, which influences the overall integrity of the airways—airway smooth muscle as the actuator, cartilage as the regulator, and epithelium as the activator. As the limelight expands on the interactome of the airways, the need arises to look deeper into these interactions to fully understand integrity maintenance and pathophysiological conditions of the airways. Such understanding can lead to well-defined ways to successfully engineer the tissue of the airways.

## 4. Implications for Tissue Engineering and Therapy Design

### 4.1. Composition and Failure Modes of Current Constructs

The systematic analysis of engineered airway constructs reveals recurring compositional deficiencies that directly correlate with clinical and preclinical failures. Typical construct compositions and their documented limitations are summarised in Table 2.

We hypothesise that the fundamental failure of engineered airway constructs lies not simply in the absence of individual cell types but in the disruption of essential mechanotransduction–secretome feedback loops that maintain airway homeostasis. Recent insights into secretory mechanotransduction in our paper reveal that airways function through a closed-loop system where mechanical forces directly regulate cellular secretory processes, creating a dynamic equilibrium essential for tissue integrity.

### 4.2. Paradigm Shift: From Cell-Centric to Systems-Level Engineering

The traditional focus is on individual cell types and material properties; it assumes that successful constructs require correct cell seeding densities and biocompatible scaffolds. Analysis of failure with these mindsets attribute it to cell loss, poor integration, or mechanical mismatch. However, a mechanotransduction-informed approach with a focus on integrated mechanotransduction–secretome feedback loops might lead to the construction of a more matured and advanced engineered tissue. This comes with this article’s core concept that functional constructs require coordinated cellular communication networks and that failures were attributed to disrupted homeostatic mechanisms and lost mechanotransduction pathways, disabling the full maturation pathway of the tissue-engineered airways. Table 3 shows the comparative engineering strategies for a novel approach for tissue engineered constructs using a mechanotransductive–secretome paradigm.

### 4.3. Translational Considerations: Animal Model Selection and Scaling Effects

Cellular mechanotransduction effects vary significantly across different airway sizes and species due to fundamental allometric scaling laws that govern anatomical, ventilatory, and physiological differences [257,258,259]. This review focuses on generalised airway mechanotransduction effects while explicitly identifying whether data sources originate from mice, human, or porcine studies to address these species-specific variations. Small mammalian models, while valuable for mechanistic insights into secretome–mechanotransduction interactions, cannot replicate human-relevant mechanotransduction environments due to differences in airway geometry, ventilatory patterns, and device performance scaling [260,261,262]. Porcine airways serve as critical translational bridges, offering human-relevant anatomy and clinical-scale mechanotransduction assessment capabilities that enable realistic evaluation of tissue-engineered constructs under physiological mechanical loading conditions [262,263]. Readers should remain cognizant of these species-specific mechanotransduction pathway differences when interpreting the mechanisms underlying airway engineering applications across different experimental models [264,265].

### 4.4. Conclusions

This comprehensive review presents the first unified map of bidirectional mechanobiology–secretome interactions within human airways, revealing a novel three-component regulatory architecture where epithelial cells function as environmental activators, smooth muscle as mechanical actuators, and cartilage as calcium-dependent regulators. The framework identifies critical mechanotransduction pathways—particularly YAP/TAZ signalling, TRPV4 channels, and TGF-β activation—that create closed-loop feedback systems linking matrix stiffness to cytokine release and pathological airway remodelling [61,266].

As the field advances toward comprehensive airway interaction mapping, we advocate for a fundamental paradigm shift that places mechanobiology at the centre of experimental design and data interpretation. Researchers must abandon the traditional reductionist approach of studying isolated cell types in static culture conditions and instead embrace mechanobiology-aware systems thinking that recognises the airway as an inherently mechanical organ where cellular communication is inseparable from physical forces [267]. We strongly recommend that future studies integrate spatial multi-omics platforms with real-time mechanotransduction monitoring, combining single-nucleus RNA sequencing and spatial transcriptomics (Visium) with simultaneous measurement of YAP/TAZ localization, TRPV4 activation states, and matrix remodelling dynamics across anatomical niches [268,269]. The research community should prioritise organoid-based interaction mapping using lamination-based spatially resolved transcriptomics (LOSRT) that preserves tissue architecture while enabling mechanical conditioning through breath-mimicking cyclic stretch and controlled matrix stiffness gradients [270]. Critically, we believe researchers must move beyond correlational network analysis toward causal mechanistic frameworks that employ targeted perturbation experiments—using pharmacological inhibitors (verteporfin for YAP/TAZ, TRPV4 modulators), mechanical interventions (substrate stiffness manipulation), and secretome modulation (MSC-derived exosomes)—to establish directional signalling relationships rather than mere associations. The future success of airway interaction research depends on standardising mechanotransduction-inclusive experimental protocols where every study incorporates physiological mechanical stimuli as a fundamental variable, recognising that the mechanical environment governs not only individual cell behaviour but the entire landscape of intercellular communication networks that maintain airway homeostasis and drive disease progression.

In application for airway engineering, the mechanotransduction-informed approach represents a fundamental paradigm shift from component-based to systems-based airway tissue engineering. By understanding and recreating the bidirectional mechanotransduction–secretome feedback loops essential for airway homeostasis, this approach addresses the root causes of engineering failures rather than their symptoms. This approach transforms airway tissue engineering from empirical optimisation to mechanistically informed design, offering significantly improved prospects for clinical success.

## Figures and Tables

**Table 2:** Common airway construct compositions and associated failure modes.

Scaffold Type	Typical Cellularization	Key Limitations	References
Synthetic polymer scaffolds (PCL, PLA)	Epithelial cells, MSCs, chondrocytes	Variable epithelial coverage; mechanical mismatch causing stenosis	[252,253]
Decellularized tracheal grafts	Epithelial cells + MSCs/ chondrocytes	Higher stenosis rates with single-cell seeding; contamination risk	[252,253]
3D-bioprinted hydrogels	Airway epithelial progenitors, MSC-derived SMCs	Mechanical collapse under contractile load; immature SMC phenotype	[254,255]
Composite biomaterials (silk-collagen)	hiPSC-derived epithelial patches	Short-term integration only; long-term durability unproven	[256]

**Table 3:** Comparative analysis of engineering strategies.

Cell Selection and Differentiation		
**Aspect**	**Traditional Strategy**	**Mechanotransduction-Informed Strategy**
Cell Sources	Primary cells, basic iPSC differentiation	iPSCs engineered for enhanced mechanotransduction (TRPV4, YAP/TAZ)
Differentiation Goals	Achieve cell type identity	Achieve functional mechanotransduction networks
Quality Metrics	Cell viability, marker expression	Mechanotransduction pathway activity, cellular specialisation roles
**Scaffold Design Philosophy**		
**Aspect**	**Traditional Strategy**	**Mechanotransduction-Informed Strategy**
Material Selection	Biocompatible polymers, decellularized matrices	Biomimetic stiffness gradients (2–50 kPa)
Mechanical Properties	Uniform stiffness matching native tissue	Spatially varied stiffness to guide YAP/TAZ activation
Design Rationale	Provide structural support	Create mechanotransduction- responsive environments
Failure Prevention	Mechanical reinforcement	Dynamic adaptation through feedback loops
**Culture and Conditioning Approaches**		
**Aspect**	**Traditional Strategy**	**Mechanotransduction-Informed Strategy**
Bioreactor Design	Static or simple dynamic culture	Multi-compartment mechanotransduction platforms
Mechanical Stimulation	Generic cyclic loading	Pathway-specific conditioning (YAP/TAZ, TRPV4, TGF-β)
Monitoring Parameters	Cell growth, basic function	Real-time mechanotransduction pathway activity
Maturation Goals	Tissue-like structure	Functional homeostatic mechanisms

## Data Availability

No new data were created or analysed in this study.

## Abbreviations

The following abbreviations are used in this manuscript:

MSC	Mesenchymal Stromal Cell
AEC	Airway Epithelial Cell
ASMC	Airway Smooth Muscle Cell
ECM	Extracellular Matrix
YAP	Yes-associated Protein
TAZ	Transcriptional Coactivator with PDZ-binding Motif
TRPV4	Transient Receptor Potential Vanilloid 4
FGF-10	Fibroblast Growth Factor 10
COPD	Chronic Obstructive Pulmonary Disease
TGF-β	Transforming Growth Factor Beta
PGE2	Prostaglandin E2
NO	Nitric Oxide
IL-1β	Interleukin 1 Beta
VEGF	Vascular Endothelial Growth Factor
CTGF	Connective Tissue Growth Factor
EGF	Epidermal Growth Factor
IGF	Insulin Growth Factor
PDK1	Three-Phosphoinositide Dependent Protein Kinase 1
ROCK	Rho-associated, coiled-coil containing protein kinase
EMT	Epithelial–Mesenchymal Transition
